# Supplementary material for: RNA splicing is a key mediator of tumour cell plasticity and a therapeutic vulnerability in colorectal cancer
Source: Nat Commun. 2022 May 19;13:2791. doi: 10.1038/s41467-022-30489-z (PMC9120198; doi:10.1038/s41467-022-30489-z)
Supplement: Supplementary file 1 — Supplementary Information [file 41467_2022_30489_MOESM1_ESM.docx]

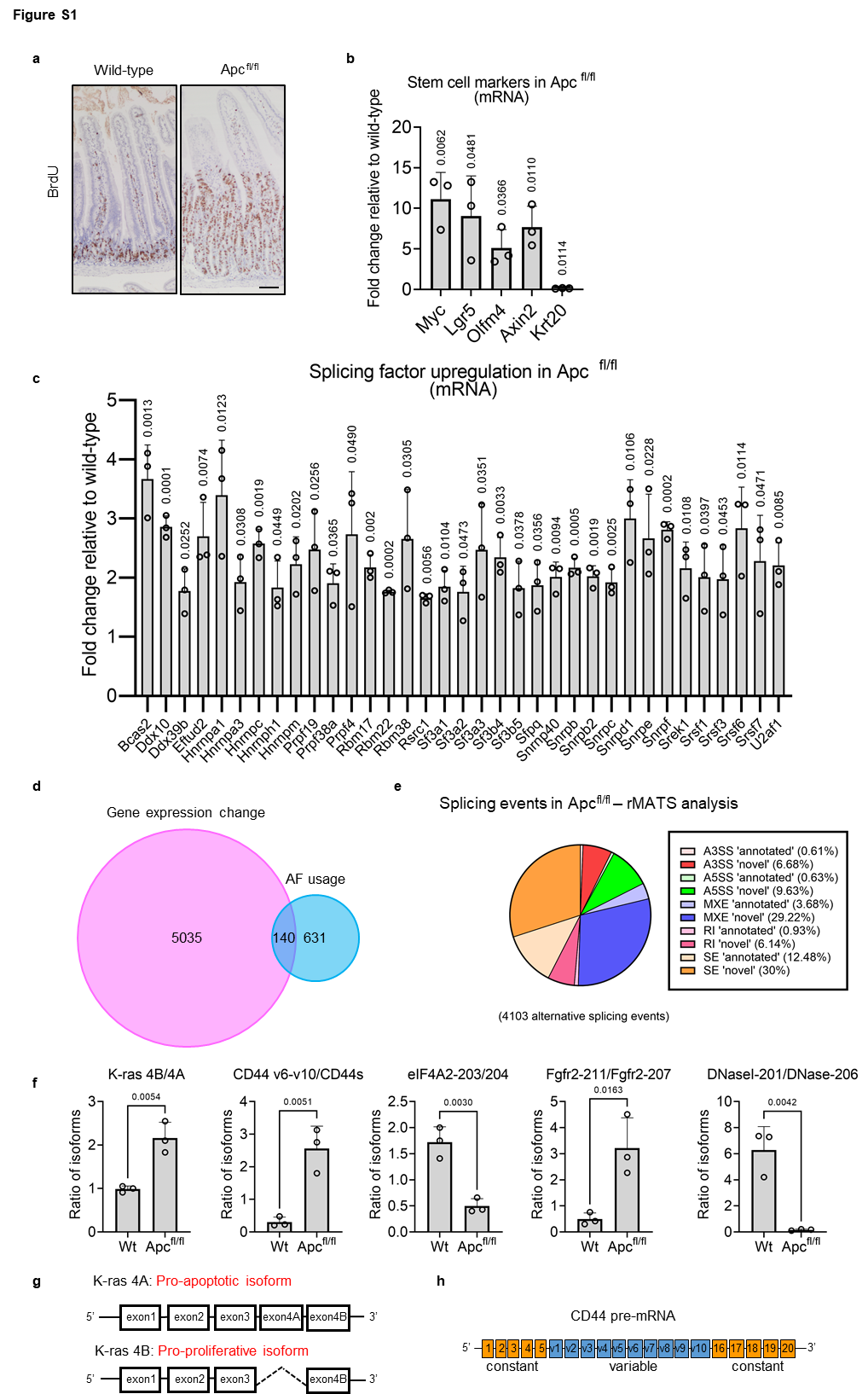


**
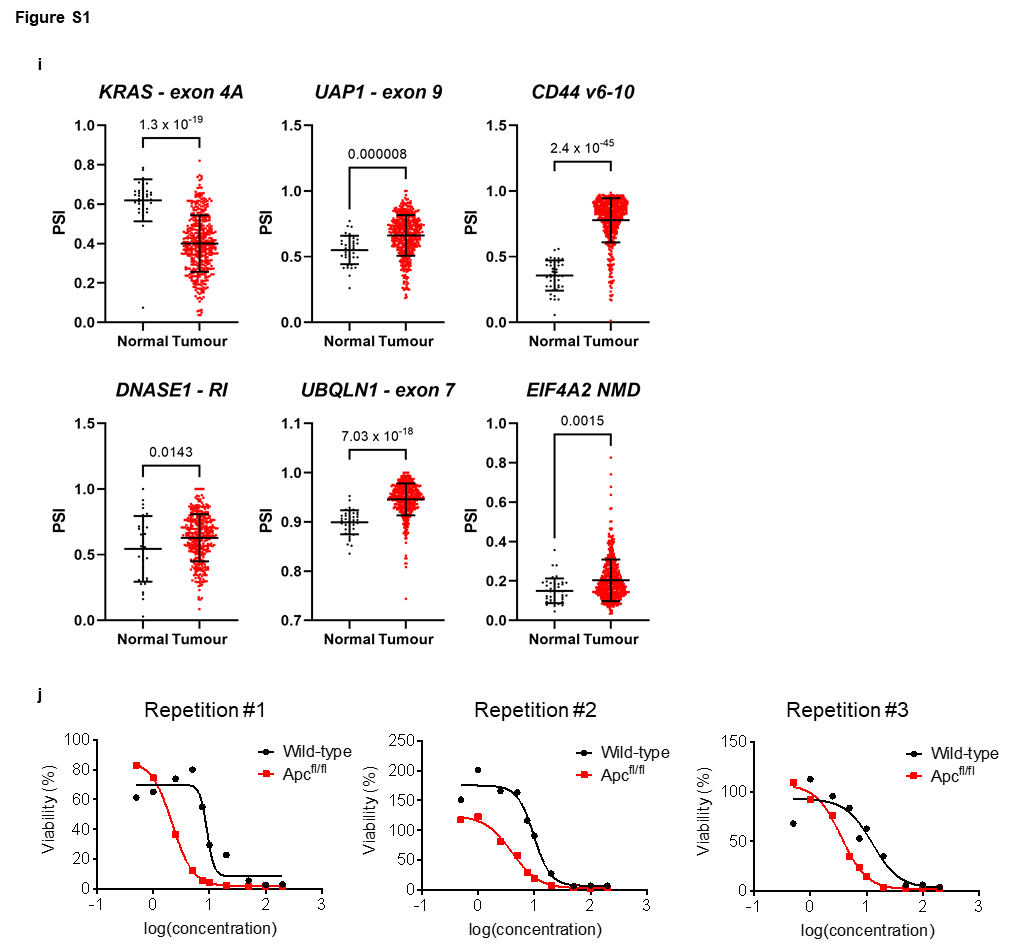
**

**Figure S1 related to Figure 1.**

(A) Representative images of wild-type or *Apc*-deleted (5 days post induction) mouse small intestines stained with anti-BrdU. Scale bar 100 µm. (B) qPCR showing fold-changes in stem cell (*Myc, Lgr5, Olfm4,* and *Axin2*) or differentiated (*Krt20*) cell markers in *Apc*^fl/fl^ small intestinal tissue relative to wild-type, normalised to the geometric mean of *Gapdh* and *18srRNA*. (C) qPCR showing fold-changes in splicing factors upregulated in *Apc*^fl/fl^ small intestinal tissue relative to wild-type normalised to the geometric mean of *Gapdh* and *18srRNA*. (D) Venn diagram of overlap between gene expression changes and alternative first exon annotated splicing events following *Apc* deletion. (E) Pie chart of splicing events identified by rMATS analysis following *Apc* deletion according to type of event and proposed ‘annotated’ or ‘novel’ designation. (F) Quantification of RT-PCR splicing isoform changes shown in Figure 1C. (G) Schematic of *Kras* splicing isoforms and their function as demonstrated previously ^17, 18^. (H) Schematic of the splicing events in CD44 as previously documented ^45-47^. (I) Individual value plots of percent spliced in (PSI) values of indicated genes in TCGA normal vs CRC tumour tissue, n = 41 vs 457 biologically independent tumours. (J) Cell viability plots (MTT) following exposure of indicated intestinal organoids to pladienolide B. All experiments n=3. Data represented as mean and error bars SD. Data (in B, C, F and I) analysed with two-tailed, unpaired t-test, p values are indicated in figure panels.


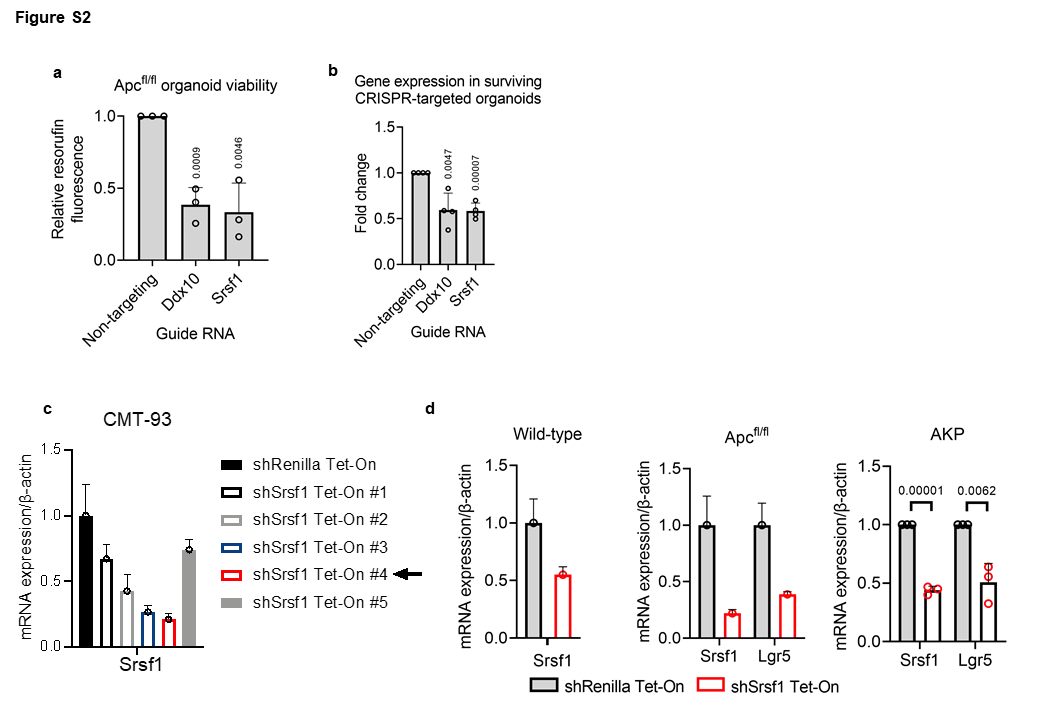


**Figure S2 related to Figure 2.**

(A) *Apc*^fl/fl^ Cas9 expressing organoid viability (resazurin) following treatment with the indicated gRNA. (B) qPCR of indicated genes (normalised to β-actin) following gRNA treatment in pooled *Apc*^fl/fl^ Cas9 organoids, n = 4 independent experiments. (C) *Srsf1* expression following control (Renilla) or *Srsf1* shRNA treatment (5 different sequences) in CMT-93 2D cells. (D) qPCR quantification of indicated genes in wild-type, *Apc*^fl/fl^ and AKP organoids using *Srsf1* shRNA#4. All data are n=3 except wild-type and *Apc*^fl/fl^ qPCR in (D) which are n=1 with 3 technical replicates. Two-tailed, unpaired t-test used for all and data are represented as mean with error bars showing SD (panels A and B) or SEM (panel C, and D), p values are indicated in figure panels.

**
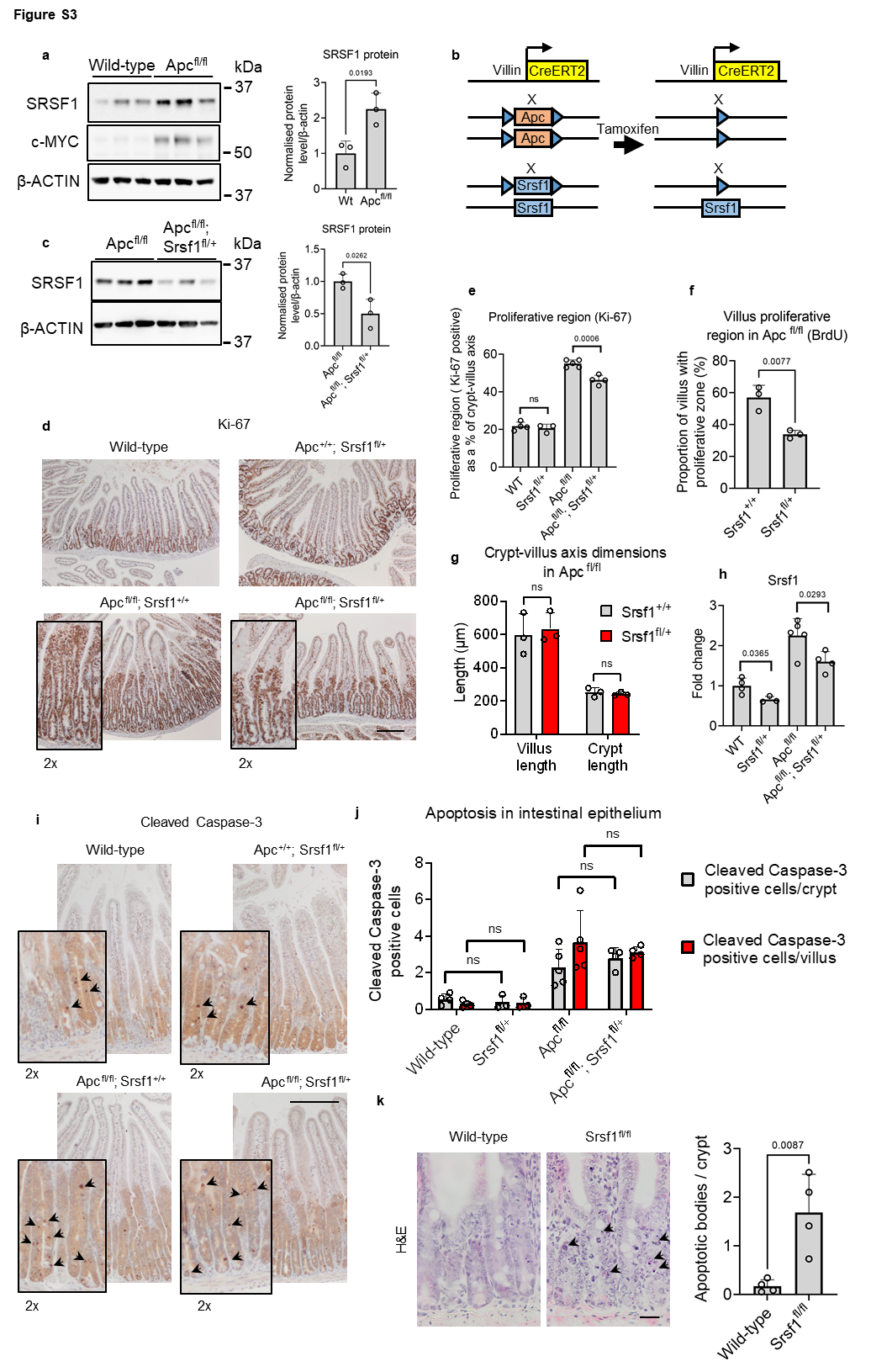

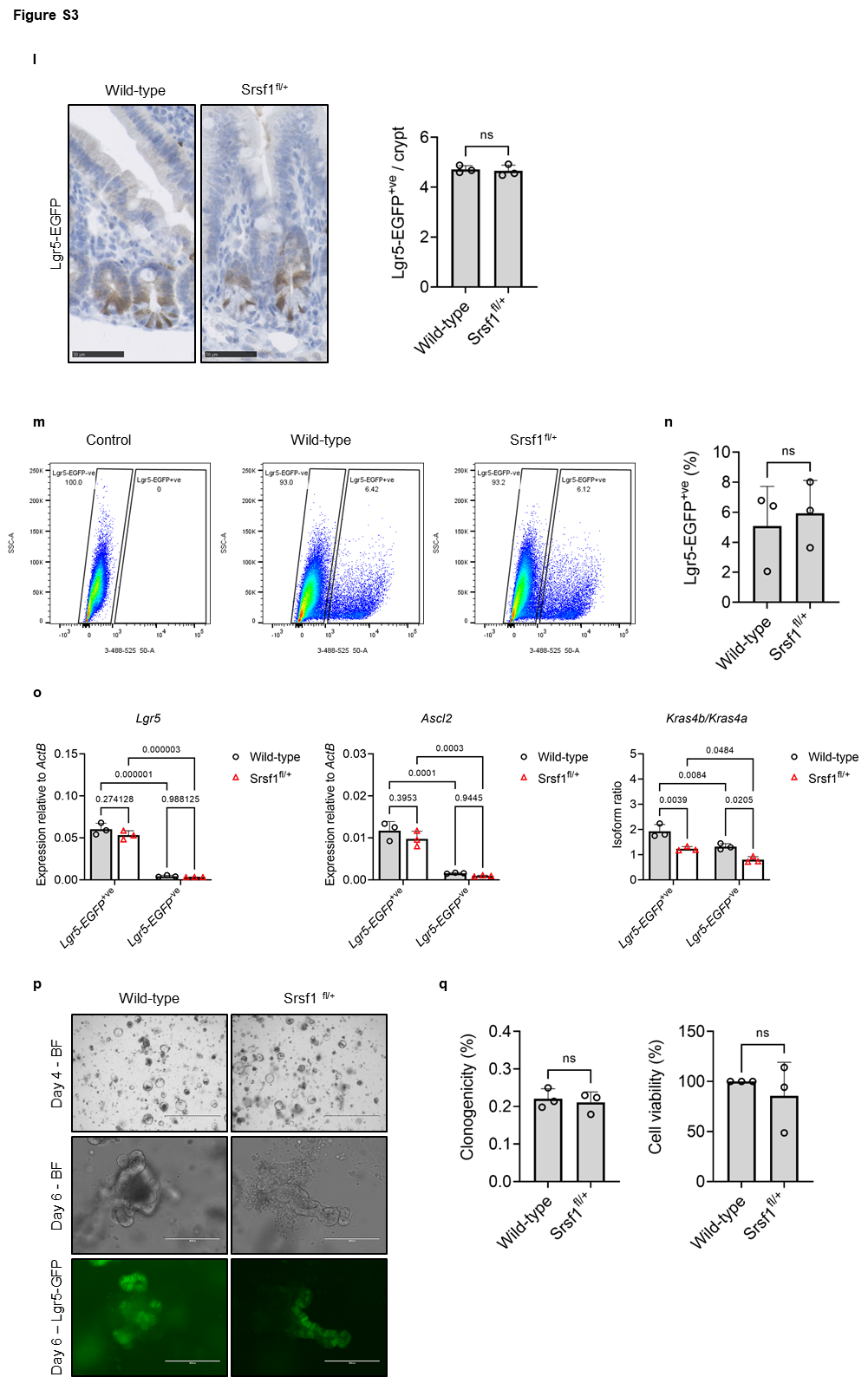

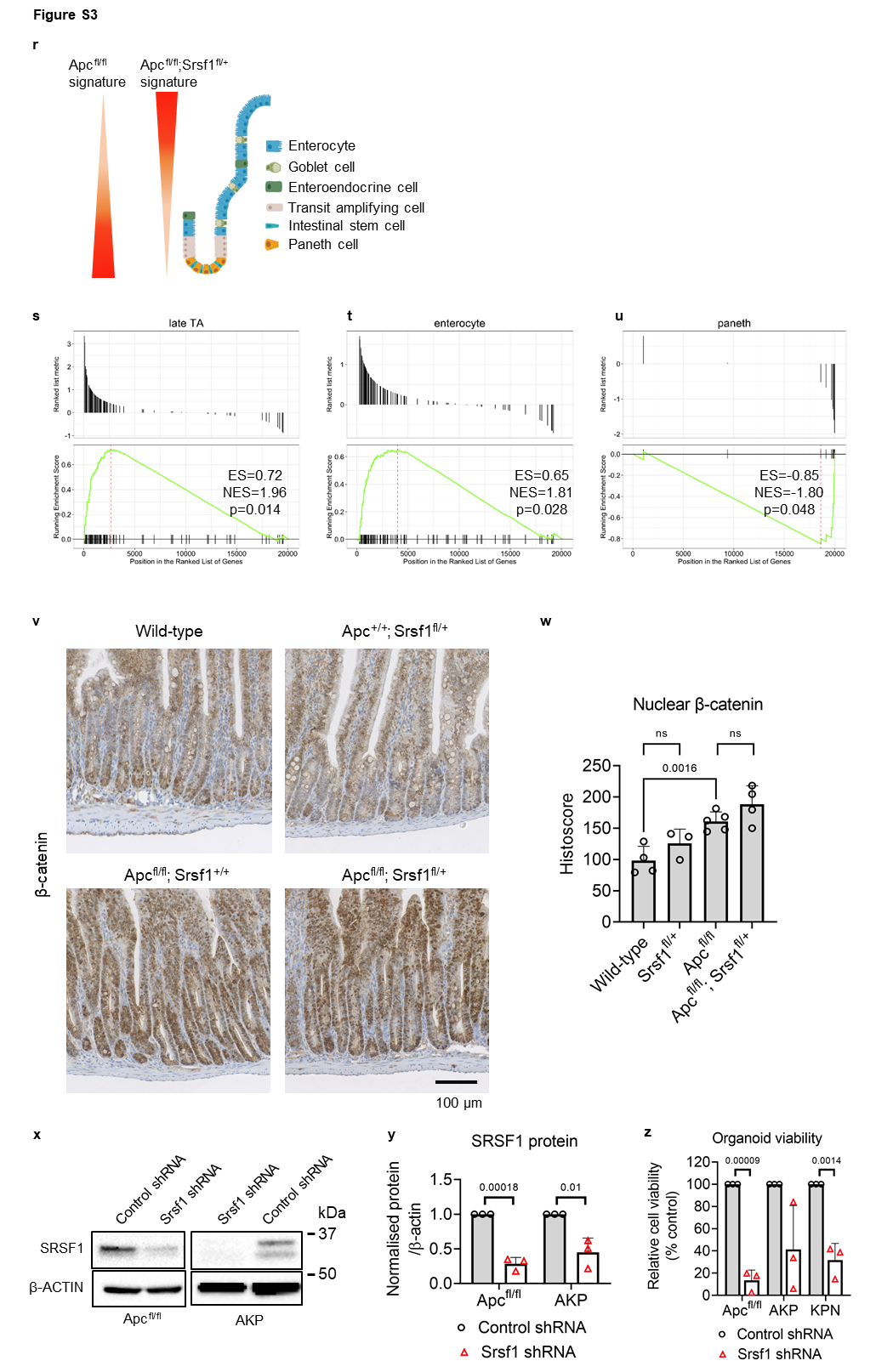
**

**Figure S3 related to Figure 3.**

(A) Western blots using lysates from wild-type and *Apc*^fl/fl^ mouse small intestines, and quantification of SRSF1 protein levels, n = 3 vs 3 biologically independent replicates. (B) Schematic showing Cre-lox recombined *Apc*^fl/fl^; *Srsf1*^fl/+^ intestinal tissue following tamoxifen induction. (C) Western blots using lysates from *Apc*^fl/fl^ and *Apc*^fl/fl^; *Srsf1*^fl/+^ mouse small intestines, and quantification of SRSF1 protein levels, n = 3 vs 3 biologically independent replicates. (D) Ki-67 staining of mouse small intestines with indicated genotypes. 5 days post induction. Scale bar 250 µm. (E) Proliferative region of small intestine following tamoxifen induction assessed by Ki-67 staining, n = 4 vs 3 vs 5 vs 4 biologically independent replicates. (F) Proportion of villi with proliferative cells assessed by BrdU staining, n = 3 vs 3 biologically independent replicates. (G) Assessment of crypt and villus dimensions in the indicated genotypes, n = 3 vs 3 biologically independent replicates. (H) *Srsf1* transcript levels (qPCR), normalised to β-actin. (I) Active (cleaved) caspase-3 staining in the small intestines of mice from the indicated genotypes. 5 days post induction. Scale bar 250 µm. (J) Apoptosis quantification in small intestines assessed by cleaved caspase-3 staining, n = 4 vs 3 vs 5 vs 4 biologically independent replicates. (K) H&E staining of intestine from wild-type and *Srsf1^fl/fl^* intestine. 5 days post induction. Black arrows indicate apoptotic bodies (left panels). Scale bar 50 µm. Quantification of apoptotic bodies (right panel), n = 4 vs 4 biologically independent replicates. (L) GFP IHC to detect Lgr5-GFP cells in wild-type and *Srsf1^fl/+^* small intestine (left panels. Quantification of Lgr5-EGFP positive cells per crypt (right panel), n = 3 vs 3 biologically independent replicates. 7 days post induction. (M) FACs plots showing gating strategy for sorting Lgr5+ intestinal stem cells. (N) Quantification of percentage positive Lgr5-GFP cells in wild-type and *Srsf1^fl/+^* intestines, n = 3 vs 3 biologically independent replicates. (O) QRT-PCR analysis of various intestinal stem cell markers in sorted Lgr5-GFP +ve and Lgr5-GFP –ve cells derived from wild-type and *Srsf1^fl/+^* intestines, n = 3 vs 3 biologically independent replicates. (P) Representative images of colonies formed from plated single Lgr5-GFP +ve cells from wild-type and *Srsf1^fl/+^* intestines. Scale bars are 1000 µm (top panels) and 250 µm (middle and bottom panels). (Q) Quantification of clonogenicity experiments, n = 3 vs 3 biologically independent replicates. (R) Schematic showing the change in cellular composition of the small intestine following *Srsf1* impairment. (S-U) Gene Set Enrichment Analysis (GSEA) of *Apc*^fl/fl^; *Srsf1*^fl/+^ RNAseq and different cellular gene signatures. (V) β-catenin IHC of small intestines from indicated genotypes. 5 days post induction. (W) Quantification of β-catenin IHC, n = 4 vs 3 vs 5 vs 4 biologically independent replicates. (X) Representative Western blots on indicated organoids following control or *Srsf1* shRNA treatment and (Y) quantification, n = 3 vs 3 independent experiments. (Z) Organoid viability (resazurin) in indicated organoids following control or *Srsf1* shRNA treatment, n = 3 vs 3 independent experiments. Data in bar charts are represented as mean and error bars are SD with data analysed with two-tailed, unpaired t-tests, p values are indicated in figure panels. All biological replicates are shown as individual value plots.


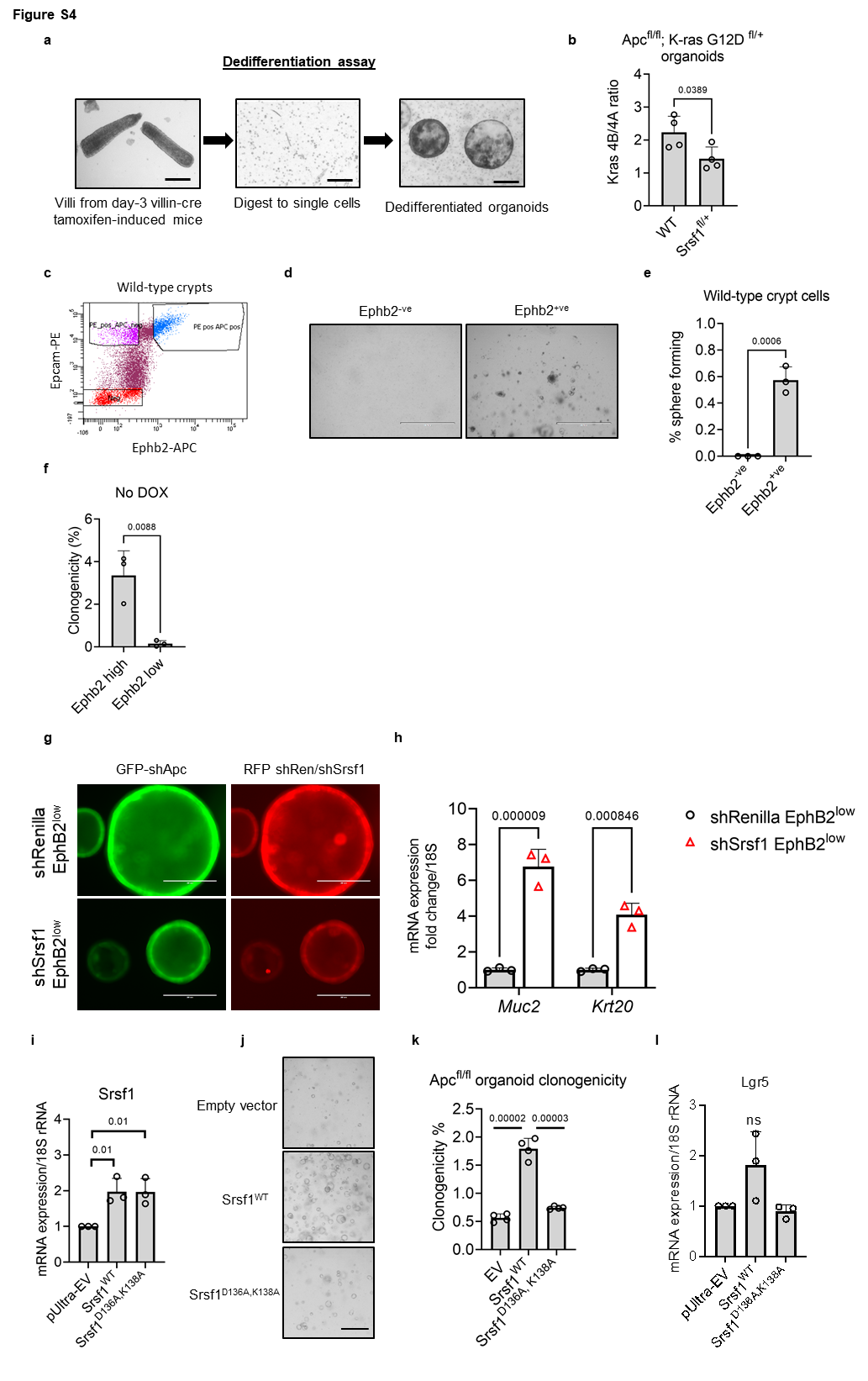


**Figure S4 related to Figure 4.**

(A) Schematic depiction of how the dedifferentiation assay was executed. Scale bars 250 µm. (B) Ratio of *Kras* splicing isoforms in *Apc*^fl/fl^; *Kras*^G12D fl/+^ organoids as determined by qPCR, n = 4 vs 4 independent experiments. (C) FACs plot outlining gating strategy for sorting Epcam-PE positive / Ephb2-APC negative cells from wild-type crypts. (D) Representative images of colonies formed from Ephb2 negative and positive cells. Scale bars 1000 µm (E) Quantification of clonogenicity assays, n = 3 vs 3 biologically independent experiments. (F) Quantification of clonogenicity assays of Ephb2^high^ and Ephb2^low^ cells derived from the *shApc, Kras^G12D^* colonic organoid model prior to doxycycline induction, n = 3 vs 3 independent experiments. (G) Representative images of organoids derived from doxycycline induced *shApc, Kras^G12D^* colonic organoids expressing either *shRenilla* or *shSrsf1.* Scale bars 250 µm. (H) QRT-PCR analysis of various markers of differentiated intestinal cells in these organoid models, n = 3 vs 3 independent experiments. (I) *Srsf1* levels (qPCR) in *Apc*^fl/fl^ organoids where either wild-type *Srsf1* (*Srsf1*^WT^) or *Srsf1* with a forced mutation in the second RNA recognition motif (RRM2) (*Srsf1*^D136A,K138A^) were constitutively overexpressed, or empty vector (EV) control, n = 3 vs 3 independent experiments. (J) Images from clone-forming assay in *Srsf1*^D136A,K138A^ expressing *Srsf1*^WT^ or *Srsf1*^D136A,K138A^. Scale bar is 500μm. (K) Quantification of clonogenicity, n = 4 vs 4 vs 4 independent experiments. (L) *Lgr5* levels (qPCR) in indicated organoids, n = 3 vs 3 independent experiments. Two-tailed, unpaired t-test used, p values are indicated in figure panels. Data are represented as mean with error bars showing SD.


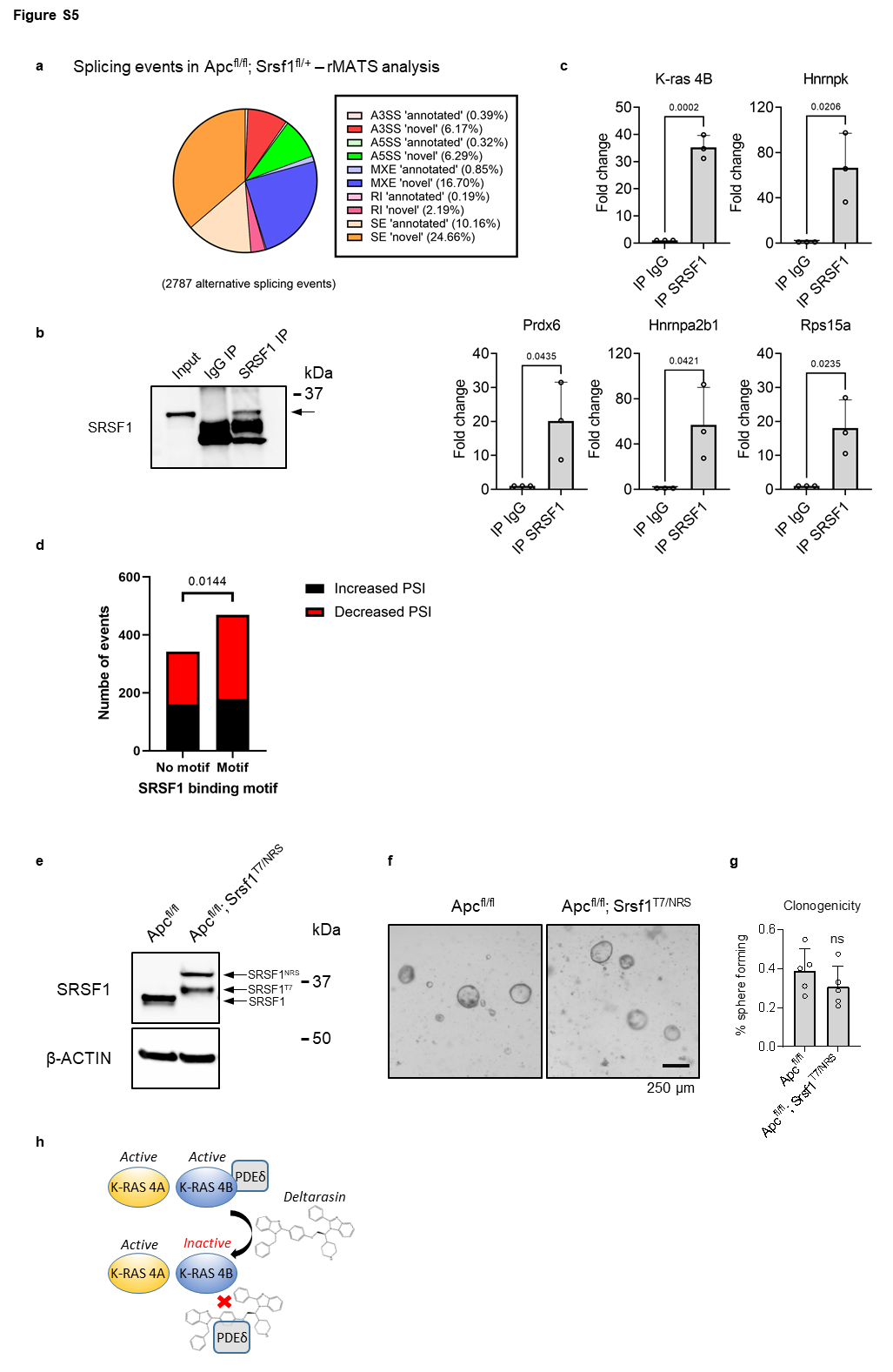

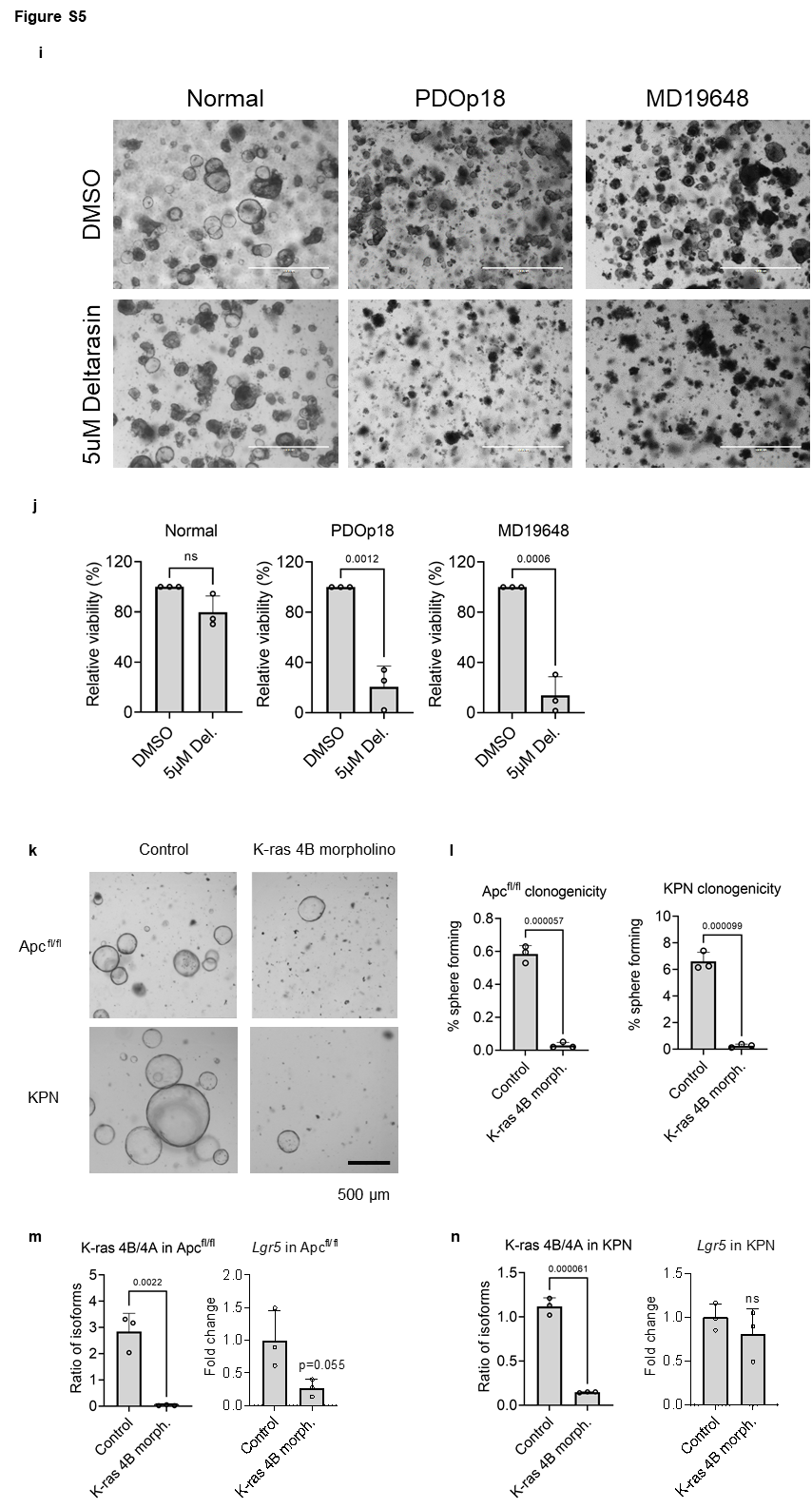

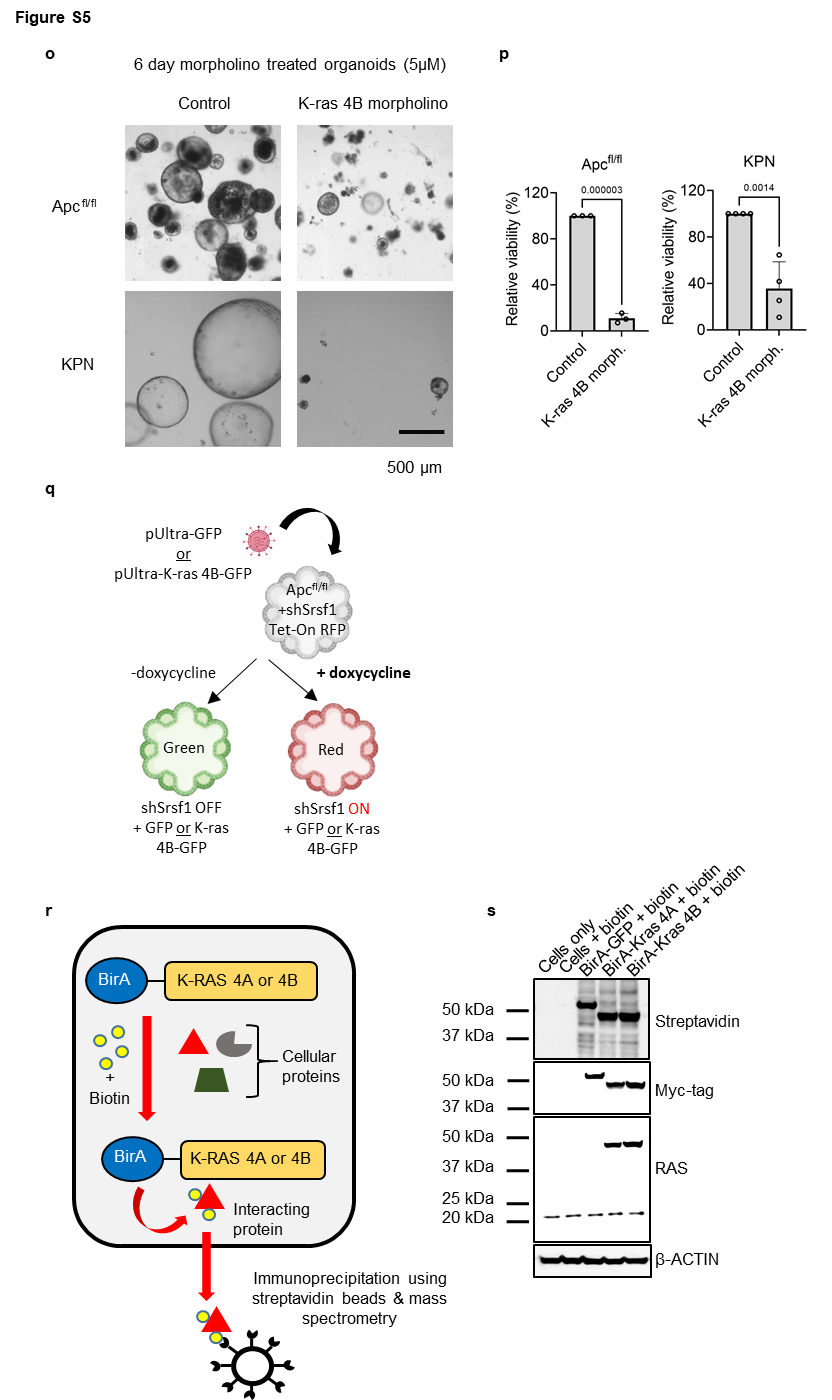

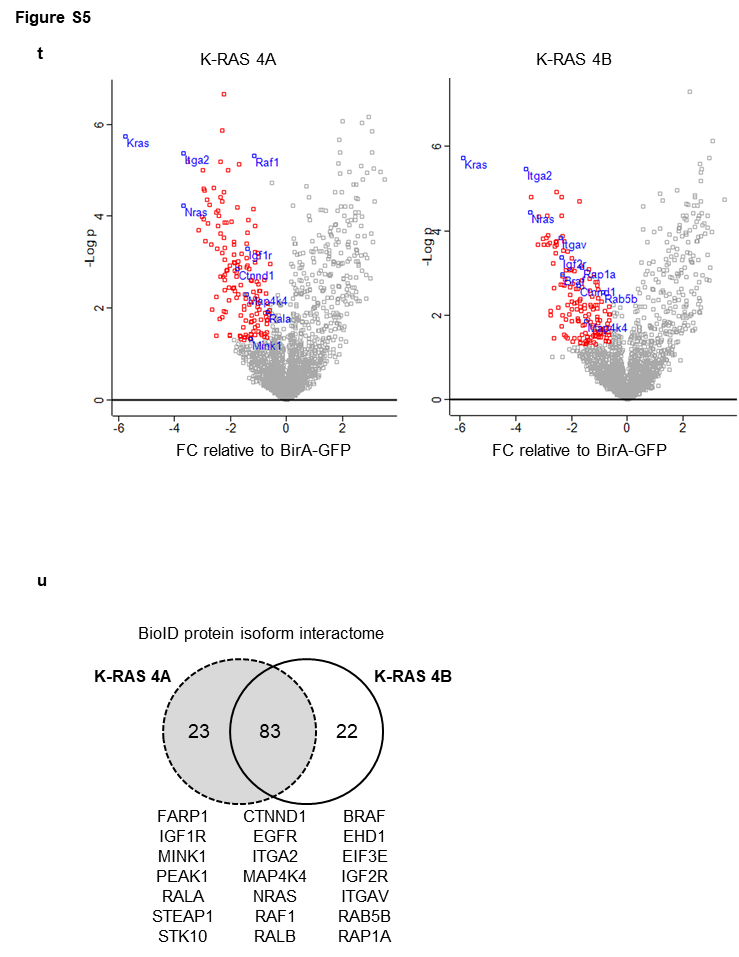


**Figure S5 related to Figure 5.**

(A) Pie chart of splicing events identified by rMATS analysis following *Apc* and *Srsf1^fl/+^* deletion according to type of event and proposed ‘annotated’ or ‘novel’ designation. 5 days post induction. (B) Western blot of SRSF1 IP from CMT93 cells. (C) QRT-PCR analysis of transcripts identified as alternatively spliced following *Srsf1* depletion following control and SRSF1 RNA IP experiments, n = 3 independent experiments. (D) Enrichment analysis for *Srsf1* binding motifs in exons either decreased or increased following *Srsf1* depletion. Fishers exact test, two-sided. (E) Western blot analysis of organoids derived from *Apc^fl/fl^* and *Apc^fl/fl^ Srsf1^NRS^* mice. (F) Representative images of clonogenicity experiments on organoids derived from *Apc^fl/fl^* and *Apc^fl/fl^ Srsf1^NRS^* mice. Scale bar is 250 μm. (G) Quantification of clonogenicity experiments, n = 5 independent experiments. (H) Schematic showing how deltarasin inhibits the function of KRAS4B but not KRAS4A. (I) Representative images of human normal and tumour colonic organoids treated with 5μM deltarasin. Scale bar is 1000 μm. (J) Relative viability of different organoid lines following deltarasin treatment, n = 3 independent experiments. (K) Representative images of *Apc^fl/fl^* and KPN clonogenicity experiments following treatment with *Kras4b* splice targeting antisense morpholino. Scale bar is 500 μm. (L) Quantification of clonogenicity experiments, n = 3 independent experiments. (M) QRT-PCR analysis of *Kras4b/4a* splice ratio following morpholino treatment in *Apc^fl/fl^* organoids, n = 3 independent experiments. (N) QRT-PCR analysis of *Kras4b/4a* splice ratio following morpholino treatment in KPN organoids, n = 3 independent experiments. (O) Representative images following *Kras4b* targeting morpholino treatment of preformed organoids of indicated genotypes. (P) Relative viability of morpholino treated organoids. Apc, n = 3 independent experiments, KPN, n =4 independent experiments. (Q) Schematic depiction showing experimental strategy to investigate if overexpressing *Kras4b* could rescue organoids with impaired *Srsf1* expression. (R) Schematic depiction of how proximity-dependent biotin identification (BioID) to uncover interacting proteins works and how it was used to discover the protein interactome of KRAS4A and KRAS4B. (S) Western blots of lysates from CMT-93 cells expressing different KRAS protein isoforms or GFP control, all with N-terminal biotin ligase BirA. Cells were cultured alone +/- biotin, or were cultured with the indicated vector in the presence of biotin. (T) Volcano plot showing significant (p<0.05, n=3) protein interactions of the KRAS4A and KRAS4B proteins. (U) Venn diagram showing results from the KRAS4A/4B protein interactome study (BioID) done in mouse rectal carcinoma cell line CMT-93. A selection of significantly (p<0.05, n=3) interacting proteins is shown below each set. Data in bar charts C, G, J, L, M, N and P are represented as mean and error bars are SD. Data in C, G, J, L, M, N, P and T analysed with two-tailed, unpaired t-tests, p values are indicated in figure panels. All biological replicates are shown as individual value plots.


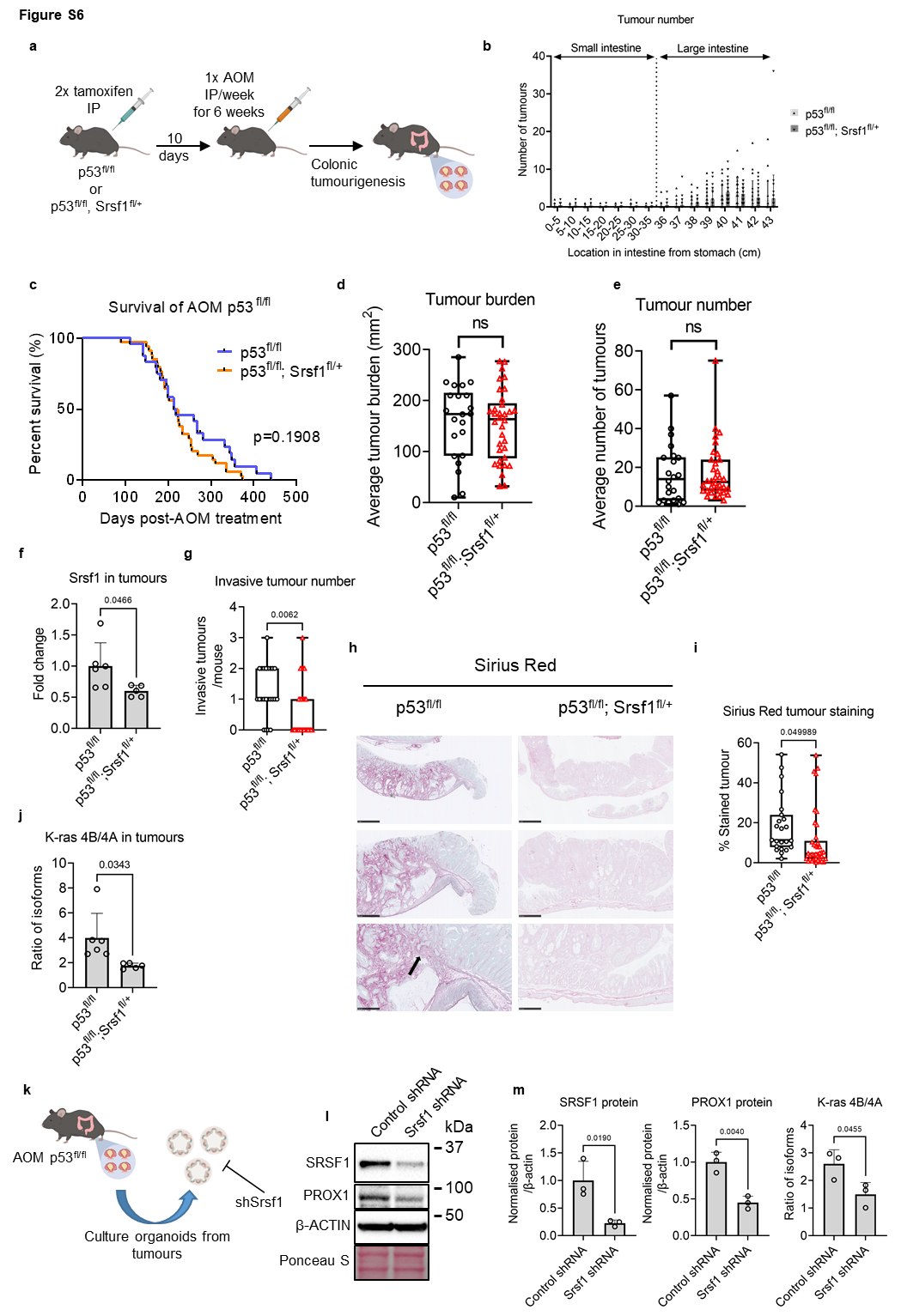


**Figure S6 related to Figure 6.**

(A) Schematic depiction of the advanced tumour mouse model, showing tamoxifen-induced Cre-lox deletion of *Tp53* alone or with *Srsf1*^fl/+^, followed by repeated treatment with the azoxymethane (AOM) carcinogenic compound. (B) Distribution of the number of tumours in the indicated AOM-treated mouse genotypes across the intestine, measured from the stomach to the anus in cm. p53^fl/fl^ n=23, p53^fl/fl^; *Srsf1*^fl/+^ n=34 biologically independent mice. (C) Kaplan-Meier curves showing survival of AOM treated p53^fl/fl^ (n=24) and p53^fl/fl^; *Srsf1*^fl/+^ (n=34) biologically independent mice. Log-rank (Mantel-Cox) test shown. (D) Tumour burden and (E) tumour number in mice with the two indicated genotypes after AOM treatment and ageing, n = 22 vs 34 biologically independent mice. (F) *Srsf1* levels as determined by qPCR in dissected tumours from the indicated mice (n>5), normalised to β-*actin*, n = 6 vs 5 biologically independent tumours. (G) Number of individual invasive tumours for each mouse of the indicated genotype, n = 23 vs 34 biologically independent mice. (H) Representative histological images of mouse intestines and tumours stained with Sirius red. Scale bars are 1 mm (2.5x), 500 µm (5x) and 250 µm (10x). (I) Quantification of sirius red staining (which highlights collagen) in colonic tumours from mice with the indicated genotypes after AOM treatment and ageing, n = 23 vs 38 biologically independent tumours. (J) qPCR-derived ratio of *Kras* splicing isoforms in tumours from AOM treated p53^fl/fl^ mice, n = 6 vs 5 biologically independent mice. (K) Schematic showing strategy for removal of tumours from AOM treated p53^fl/fl^ mice and subsequent organoid culture, followed by shRNA manipulation. (L) Representative Western blots following *Srsf1* targeting by shRNA in tumour-derived organoids from p53^fl/fl^ mice. (M) Quantification of Western blot staining for SRSF1 and PROX1 and qPCR of *Kras* splicing isoform ratio in *Srsf1* shRNA-manipulated p53^fl/fl^ tumour organoids, n = 3 independent experiments. Data in bar charts B, F, J and M are represented as mean and error bars are SD. In box plots D, E, G and I the box extends from 25^th^ to 75^th^ centiles, centre line is median and whiskers extend to minima and maxima. Data in (D, E, F, G, I, J and M) analysed with two-tailed, unpaired t-tests, p values are indicated in figure panels. All biological replicates are shown as individual value plots.


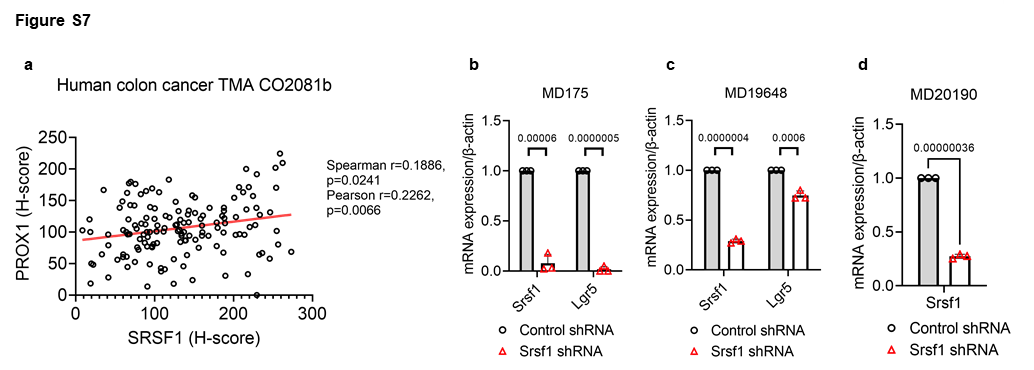


**Figure S7 related to Figure 7.**

(A) Linear regression analysis showing the correlation of SRSF1 and PROX1 staining (based on histoscore) on TMA CO2081b with each datapoint representing a core taken from a patient. (B, C and D) qPCR-determined gene expression of *Srsf1* and *Lgr5* in the indicated patient-derived organoids following shRNA treatment. Data in bar charts are represented as mean and error bars are SD with data analysed with two-tailed, unpaired t-tests, p values are indicated in figure panels. All biological replicates are shown as individual value plots and n=3 vs 3 independent replicate experiments.

**Supplementary Data legends.**

**Supplementary Data 1.** List of gene expression changes in WT vs *Apc* deficient small intestine. Log fold change, p value and multiple corrections calculated using cuffdiff statistical analysis software. Benjamini-Hochberg correction used for multiple-testing.

**Supplementary Data 2.** List of splicing factors with altered expression following *Apc* deletion.

**Supplementary Data 3.** SUPPA2 analysis of alternatively spliced transcripts following *Apc* deletion. PSI and p values calculated using SUPPA2 statistical analysis software. Benjamini-Hochberg correction used for multiple-testing.

**Supplementary Data 4.** rMATS analysis of alternatively spliced transcripts following *Apc* deletion. PSI, p values and multiple corrections calculated using rMATs statistical analysis software. FDR correction method used for multiple-testing.

**Supplementary Data 5.** List of splicing alterations identified following *Apc* deletion and whether the same alterations are detected in human colorectal cancer. PSI and p values calculated using SUPPA2 statistical analysis software.

**Supplementary Data 6.** List of gRNA sequences used for CRISPR screen.

**Supplementary Data 7.** Statistical analysis of CRISPR screen data. Log fold change, p value and multiple corrections calculated using MAGeCK statistical analysis software. Benjamini-Hochberg correction used for multiple-testing.

**Supplementary Data 8.** List of gene expression changes in *Apc* vs *Apc Srsf1^fl/+^* deficient small intestine. Log fold change, p value and multiple corrections calculated using cuffdiff statistical analysis software. Benjamini-Hochberg correction used for multiple-testing.

**Supplementary Data 9.** Gene set enrichment analysis of transcriptional changes in *Apc* vs *Apc Srsf1^fl/+^* deficient small intestine.

**Supplementary Data 10.** SUPPA2 analysis of alternatively spliced transcripts in *Apc* vs *Apc Srsf1^fl/+^* deficient small intestine. PSI and p values calculated using SUPPA2 statistical analysis software. Benjamini-Hochberg correction used for multiple-testing.

**Supplementary Data 11.** List of discordant splicing events in WT vs *Apc* deficient compared to *Apc* vs *Apc Srsf1^fl/+^* deficient small intestine. PSI and p values calculated using SUPPA2 statistical analysis software.

**Supplementary Data 12.** rMATS analysis of alternatively spliced transcripts in *Apc* vs *Apc Srsf1^fl/+^* deficient small intestine. PSI, p values and multiple corrections calculated using rMATs statistical analysis software. FDR correction method used for multiple-testing.

**Supplementary Data 13.** Kras4b and Kras4a BioID interactome data. Significantly enriched hits identified using two-tailed students t-test.

**Supplementary Data 14.** Primer sequences used in this study.
