# Supplementary material for: RNA splicing is a key mediator of tumour cell plasticity and a therapeutic vulnerability in colorectal cancer
Source: Nat Commun. 2022 May 19;13:2791. doi: 10.1038/s41467-022-30489-z (PMC9120198; doi:10.1038/s41467-022-30489-z)
Supplement: Supplementary file 3 — Description of Additional Supplementary Files [file 41467_2022_30489_MOESM3_ESM.pdf]

Description of Additional Supplementary Files

Title: Supplementary Information

Description: Supplementary Figures 1 – 7 and figure legends

Title: Supplementary Data 1

Description: RNAseq analysis of wt vs Apc small intestine.

Title: Supplementary Data 2

Description: List of splicing factors with altered expression following *Apc* deletion.

Title: Supplementary Data 3

Description: SUPPA2 RNA splicing analysis of wt vs Apc RNAseq.

Title: Supplementary Data 4

Description: rMATs RNA splicing analysis of wt vs Apc RNAseq.

Title: Supplementary Data 5

Description: Overlap of mouse and human alternative splicing events.

Title: Supplementary Data 6

Description: Sequences of gRNAs used for CRISPR screen.

Title: Supplementary Data 7

Description: Statistical analysis of CRISPR screen.

Title: Supplementary Data 8

Description: RNAseq analysis of Apc vs Apc Srsf1 small intestine.

Title: Supplementary Data 9

Description: GSEA of Apc vs Apc Srsf1 RNAseq results.

Title: Supplementary Data 10

Description: SUPPA2 RNA splicing analysis of Apc vs Apc Srsf1 RNAseq.

Title: Supplementary Data 11

Description: List of discordant RNA splicing events comparing wt vs Apc and Apc vs Apc Srsf1 RNAseq results.

Title: Supplementary Data 12

Description: rMATs RNA splicing analysis of Apc vs Apc Srsf1 RNAseq.

Title: Supplementary Data 13

Description: Kras-4a and Kras-4b BioID results.

Title: Supplementary Data 14

Description: List of primers used in this study
