# Supplementary material for: RNA splicing is a key mediator of tumour cell plasticity and a therapeutic vulnerability in colorectal cancer
Source: Nat Commun. 2022 May 19;13:2791. doi: 10.1038/s41467-022-30489-z (PMC9120198; doi:10.1038/s41467-022-30489-z)
Supplement: Supplementary file 18 — Reporting Summary [file 41467_2022_30489_MOESM18_ESM.pdf]

nature research

Corresponding author(s): Kevin Myant
Last updated by author(s): Mar 16, 2022

Reporting Summary

Nature Research wishes to improve the reproducibility of the work that we publish. This form provides structure for consistency and transparency in reporting. For further information on Nature Research policies, see our Editorial Policies and the Editorial Policy Checklist.

Statistics

For all statistical analyses, confirm that the following items are present in the figure legend, table legend, main text, or Methods section.

n/a	Confirmed	
☐	☒	The exact sample size (*n*) for each experimental group/condition, given as a discrete number and unit of measurement
☐	☒	A statement on whether measurements were taken from distinct samples or whether the same sample was measured repeatedly
☐	☒	The statistical test(s) used AND whether they are one- or two-sided<br>*Only common tests should be described solely by name; describe more complex techniques in the Methods section.*
☒	☐	A description of all covariates tested
☐	☒	A description of any assumptions or corrections, such as tests of normality and adjustment for multiple comparisons
☐	☒	A full description of the statistical parameters including central tendency (e.g. means) or other basic estimates (e.g. regression coefficient) AND variation (e.g. standard deviation) or associated estimates of uncertainty (e.g. confidence intervals)
☐	☒	For null hypothesis testing, the test statistic (e.g. *F*, *t*, *r*) with confidence intervals, effect sizes, degrees of freedom and *P* value noted<br>*Give P values as exact values whenever suitable.*
☒	☐	For Bayesian analysis, information on the choice of priors and Markov chain Monte Carlo settings
☒	☐	For hierarchical and complex designs, identification of the appropriate level for tests and full reporting of outcomes
☐	☒	Estimates of effect sizes (e.g. Cohen's *d*, Pearson's *r*), indicating how they were calculated

Our web collection on statistics for biologists contains articles on many of the points above.

Software and code

Policy information about availability of computer code

Data collection	RNAseq - Illumina HiSeq 4000, CRISPR screen sequencing - Illumina MiSeq, qPCR - BioRad CFX Connect, Histology - Nanozoomer
Data analysis	Microsoft Office Excel 365, GraphPad Prism 8.3.1, RNAseq analysis - TrimGalore 0.6.5, cutadapt 1.9.1, tophat 2.1.1 and cuffdiff 2.2.1, RNA splicing analysis - salmon v0.9.1, SUPPA2.0 and rMATS, CRISPR screen analysis - cutadapt and MAGeCK v0.5.6, g:Profiler version e98_eg45_p14_ce5b097, ImageJ 1.52p, MaxQuant-Perseus v2.0.3.0, NDP.view2 U12388-01, CellSens V3.2.

For manuscripts utilizing custom algorithms or software that are central to the research but not yet described in published literature, software must be made available to editors and reviewers. We strongly encourage code deposition in a community repository (e.g. GitHub). See the Nature Research guidelines for submitting code & software for further information.

Data

Policy information about availability of data

All manuscripts must include a data availability statement. This statement should provide the following information, where applicable:
- Accession codes, unique identifiers, or web links for publicly available datasets
- A list of figures that have associated raw data
- A description of any restrictions on data availability

The RNAseq data generated in this study have been deposited in the GEO database under accession code GSE199623 (https://www.ncbi.nlm.nih.gov/geo/query/acc.cgi?acc=GSE199623) and are freely available. All analyzed expression, sequencing and proteomic data are available as Supplementary tables S1, S2, S3, S4, S5, S6, S7, S8, S9, S10, S11, S12 and S13. All figures have associated raw data which is provided with this publication. There are no restrictions on data availability.

Field-specific reporting

Please select the one below that is the best fit for your research. If you are not sure, read the appropriate sections before making your selection.

☒ Life sciences ☐ Behavioural & social sciences ☐ Ecological, evolutionary & environmental sciences

For a reference copy of the document with all sections, see nature.com/documents/nr-reporting-summary-flat.pdf

Life sciences study design

All studies must disclose on these points even when the disclosure is negative.

Sample size

Sample sizes for each experiment are outlined in the figure legends.

For all animal experiments, n > 3 mice were used for each experimental cohort. Power analyses were carried out prior to experiments being carried out to determine the minimum number of animals required for each experiment. These analyses were informed by previous and / or preliminary experiments (for example Gudino et al Nat Comms, 2021)..

For organoid experiments, all are derived from n = 3 or n > 3 independent experiments unless otherwise stated. Sample sizes were not statistically predetermined and were based on the results of previous published experiments with these models (for example Gudino et al Nat Comms, 2021).

For RNAseq and CRISPR experiments, all are derived from n = 3 independent samples. Sample sizes were not statistically predetermined and were based on the results of previous experiments with these models (for example Gudino et al Nat Comms, 2021)..

Data exclusions

No data were excluded from analysis.

Replication

All experiments (mouse, RNAseq, QRT-PCR, Western blot, organoid etc) were replicated at least 3 times using the same experimental approach or using multiple biologically independent replicates. All replication attempts were successful.

Randomization

Mice of ages 6-12 weeks of the appropriate genotype were randomly selected, with no sex-bias, for tumourigenesis studies. All mice received the same treatment (tamoxifen induction and AOM administration). Experimental groups were determined by mouse genotype (for example P53fl/fl vs P53fl/fl Srsf1fl/+).

Blinding

Investigators were blinded to the genotype of mice when monitoring for clinical signs, when carrying out histological analysis and during data collection. IHC analysis of tumour histology was carried out using QuPath software with the investigator blinded to tumour genotype. For other experiments researchers were blinded to group allocation during data collection and analysis.

Reporting for specific materials, systems and methods

We require information from authors about some types of materials, experimental systems and methods used in many studies. Here, indicate whether each material, system or method listed is relevant to your study. If you are not sure if a list item applies to your research, read the appropriate section before selecting a response.

Materials & experimental systems

n/a	Involved in the study	
☐	☒	Antibodies
☐	☒	Eukaryotic cell lines
☒	☐	Palaeontology and archaeology
☐	☒	Animals and other organisms
☐	☒	Human research participants
☒	☐	Clinical data
☒	☐	Dual use research of concern

Methods

n/a	Involved in the study	
☒	☐	ChIP-seq
☒	☐	Flow cytometry
☒	☐	MRI-based neuroimaging

Antibodies

Antibodies used

The following antibodies were used for IHC: BrdU (BD Biosciences, 347580, pH6, 1/500), PROX1 (R&D systems, AF2727, pH6, 1/100 for human tissue array, 1/200 for mouse sections), SLC13A2 (Atlas antibodies, HPA014963, pH8, 1/100), SRSF1 (Invitrogen/Thermo Fisher Scientific, 32-4600, pH8, 1/10,000), Ki67 (Abcam, ab15580, pH6, 1/2000), Active Caspase-3 (R&D systems, AF835, pH6, 1/800). Secondary detection was achieved using Dako EnVision+/HRP rabbit/mouse System, neat (Agilent technologies, K400311-2), except for PROX1 where Rabbit anti-Goat IgG was used, 1/200 (Thermo Fisher Scientific, 81-1620).
The following antibodies were used for flow cytometry: EphB2-APC conjugated antibody, 1/200 (BD Bioscience, Clone 2H9), Epcam-PE conjugated antibody, 1/200 (BD Biosciences, 563477).
The following antibodies were used for Western blot: β-actin, 1/5,000 (Cell Signalling Technology, 4970), SF2/SRSF1, 1/1,000 (Abcam, ab133689), c-Myc, 1/1,000 (Cell Signalling Technology, 9402), Prox1, 1 μg/mL (R&D systems, AF2727), Ras (27H5), 1/1,000 (Cell

	Signalling Technology, 3339), Myc-Tag (9B11), 1/10,000 (Cell Signalling Technology, 2276), and Streptavidin HRP, 1/10,000 (Abcam, ab7403). Secondary antibodies used were: Anti-Rabbit IgG HRP-linked, 1/1,000 (Cell Signalling Technology, 7074), Anti-Mouse IgG HRP-linked, 1/1,000 (Cell Signalling Technology, 7076) and Rabbit anti-Goat IgG , 1/1,000 (Thermo Fisher Scientific, 81-1620).
Validation	BrdU (BD Biosciences, 347580): Validated by absence of signal in tissue derived from mice not injected with BrdU. Validated in numerous publications (for example PMID: 3582069, PMID: 33879799)<br>Prox1 (R&D systems, AF2727): Validated on manufacturers website by Western blot analysis of lysates of HepG2 human hepatocellular carcinoma cell line.<br>Slc13a2 (Atlas antibodies, HPA014963): Validated on manufacturers website by immunohistochemistry analysis in human duodenum and liver tissues.<br>Srsf1 (Invitrogen/Thermo Fisher Scientific, 32-4600): Validated on manufacturers website by immunofluorescence analysis of MCF-7 cells.<br>Ki67 (Abcam, ab15580): Validated on manufacturers website by immunofluorescence analysis of rabbit retina.<br>Active Caspase-3 (R&D systems, AF835): Validated on manufacturers website by immunohistochemistry analysis of human colon cancer tissue.<br>β-actin (Cell Signalling Technology, 4970): Validated on manufacturers website by Western blot analysis of cell extracts from various mouse and human cell lines.<br>SF2/SRSF1 (Abcam, ab133689): Validated on manufacturers website by Western blot in various mouse and human cell line extracts.<br>c-Myc (Cell Signalling Technology, 9402): Validated on manufacturers website by Western blot analysis of extracts from HeLa cells 48 hours following mock transfection, transfection with nonspecific (control) siRNA or transfection with c-Myc siRNA.<br>Ras (Cell Signalling Technology, 3339): Validated by absence of signal in protein lysate derived from cells not transfected with Ras overexpression vector. Also by Western blot analysis of extracts from 293T, C2C12 and C6 cells<br>Myc-Tag (Cell Signalling Technology, 2276): Validated for Western blotting by absence of signal in protein lysate derived from cells transfected or not transfected with Myc-tagged protein.<br>Streptavidin HRP (Abcam, ab7403): Validated by reduced signal in protein lysate derived from cells not expressing BirA-tagged protein.<br>EphB2-APC conjugated antibody (BD Bioscience, Clone 2H9): Validated on manufacturers website for FACs analysis of human tissue and for use in mouse tissue in multiple publications (for example PMID: 21419747).<br>Epcam-PE conjugated antibody (BD Biosciences, 563477): Validated on manufacturers website for FACs analysis of mouse tissue.

Eukaryotic cell lines

Policy information about cell lines

Cell line source(s)	All organoid lines were derived from mice / CRC patients during the course of this study. CMT93 cell line was kindly provided by Dr Susan Farrington (IGMM, Edinburgh). HEK293 cell line was kindly provided by Dr Juan Carlos Acosta (IGMM, Edinburgh). Both cell lines originally obtained from ATCC.
Authentication	Cells were not authenticated independently.
Mycoplasma contamination	Cell lines and organoid cultures were routinely tested for Mycoplasma contamination and found to be negative.
Commonly misidentified lines (See ICLAC register)	Not used.

Animals and other organisms

Policy information about studies involving animals; ARRIVE guidelines recommended for reporting animal research

Laboratory animals	Mice were bred at the animal facilities of the University of Edinburgh and were kept in 12 h light–dark cycles and were given access to water and food ad libitum. Mice were maintained in a temperature- (20–26 °C) and humidity- (30–70%) controlled environment. Colonies had a mixed background (50% C57Bl6J, 50% S129). The genetic alleles used for this study were as follows: villinCreER, Apc (floxed), ASF/SF2 (Srsf1 floxed), Kras (G12D), IKK2ca, p53 (floxed). Both genders of mice were used for all experiments at an age of between 6 and 12 weeks once they had reached a minimum weight of 20 g. At experiment endpoints, mice were humanely sacrificed by cervical dislocation (CD) in line with UK Home Office regulations.
Wild animals	Not used in this study.
Field-collected samples	Not used in this study.
Ethics oversight	All animal experiments were performed in accordance with a UK Home Office licence (Project License 70/8885), and were subject to review by the animal welfare and ethics board of the University of Edinburgh

Note that full information on the approval of the study protocol must also be provided in the manuscript.

Human research participants

Policy information about studies involving human research participants

Population characteristics	This study did not involve human research participants but utilised human derived biospecimens to generate primary organoids. Researchers were blinded to the identification of donors. Organoids were derived from tumours with the following characteristics:

	MD175 is a 50yr old female with familial adenomatous polyposis who previously underwent a colectomy and ileorectal anastomosis who then developed a stage 2 rectal cancer - individual polyp used in this study.<br>MD20043 is an 81yr old male with a rectal cancer, TNM (T3, 2, 1).<br>MD19648 is a 45yr old female with familial adenomatous polyposis complicated by a stage 3 rectal cancer - FAP rectum tumour, TNM (pT1, pN1a, 0).<br>MD20853 is a 71yr old male with a tubulovillous adenoma with low grade dysplasia.<br>MD20910 is a 60yr old male with rectal cancer, TNM (pT2, N1b, 0).
Recruitment	Patients were recruited as having primary colorectal tumours in order to derive tumour organoids to investigate mechanisms important for this disease. No biases were present that might likely impact results.
Ethics oversight	Ethical approval for human CRC organoid derivation was carried out under NHS Lothian Ethical Approval Scottish Colorectal Cancer Genetic Susceptibility Study 3 (SOCCS3) (REC reference: 11/SS/0109). All patients provided fully informed consent for use of their tissues.

Note that full information on the approval of the study protocol must also be provided in the manuscript.
